# Identification of Cerebrospinal Fluid MicroRNAs Associated With Leptomeningeal Metastasis From Lung Adenocarcinoma

**DOI:** 10.3389/fonc.2020.00387

**Published:** 2020-04-03

**Authors:** Zhenyu Pan, Guozi Yang, Hua He, Pengxiang Gao, Tongchao Jiang, Yong Chen, Gang Zhao

**Affiliations:** ^1^Department of Neuro-Oncological Surgery, The First Hospital of Jilin University, Changchun, China; ^2^Department of Radiation-Oncology, The First Hospital of Jilin University, Changchun, China; ^3^VA Palo Alto Health Care System, Stanford University Medical School, Palo Alto, CA, United States; ^4^Cancer Center, The First Hospital of Jilin University, Changchun, China

**Keywords:** leptomeningeal metastasis, cerebrospinal fluid, microRNA profiling, lung adenocarcinoma, bioinformatic analysis

## Abstract

**Background:** Leptomeningeal metastasis (LM) has frequently been observed in patients with lung adenocarcinoma. So far, its diagnosis and disease course monitoring are still extremely difficult. Moreover, there is no effective treatment regimen for LM due to a lack knowledge on the molecular mechanism of LM. This study aimed to identify LM-related cerebrospinal fluid (CSF) miRNAs, which have potential value for diagnosing and monitoring LM and exploring the molecular mechanism.

**Methods:** CSF miRNAs were screened and verified by microarray analysis and quantitative real-time PCR (qRT-PCR) in LM patients with lung adenocarcinoma and non-LM controls, and the diagnostic performance of candidate miRNAs was evaluated. Then, candidate miRNAs in matched CSF samples from LM patients at diagnosis, after initial therapy, at relapse, and after salvage therapy, were analyzed to assess the relationship between CSF miRNAs and LM disease course. The effect of candidate miRNAs on proliferation, invasion, and migration of lung adenocarcinoma cell lines was assessed. The targeted genes of the candidate miRNA were predicted by TargetScan, miRDB, and miRTarbase online analysis tools. Gene ontology (GO) and Kyoto Encyclopedia of Genes and Genomes (KEGG) were used to analyze the functional categories of predicted target genes.

**Results:** CSF miR-7975, miR-7977, and miR-7641 were screened and verified to be statistically significantly up-regulated in LM patients compared to non-LM controls. The three miRNAs, when combined, exhibited optimal diagnostic performance. Longitudinal data of CSF miR-7975 and miR-7977 correlated well with clinical courses of LM. Overexpression of miR-7977 promoted proliferation, migration, and invasion of lung adenocarcinoma cells. Moreover, 385 targeted genes of miR-7977 were predicted and were involved in various pathways related to cancer metastasis.

**Conclusions:** This study offers insights for future research of CSF miRNAs as robust tools for diagnosing and monitoring LM. It also reveals a novel pathway for exploration of underlying mechanisms of LM.

## Introduction

Leptomeningeal metastasis (LM) is known as a devastating cancer complication that occurs when malignant cells disseminate to leptomeninges and cerebrospinal fluid (CSF) compartments (1–3). About 10% of patients with metastatic cancer suffer from LM during their course of the disease (4). Lung cancer is one of the most common causes of LM (5), of which adenocarcinoma accounts for 84–96% (6). Incidence is increasing in lung adenocarcinoma patients because of improved survival from new molecular targeted therapies (7). However, the diagnosis and disease course monitoring for LM are still challenging. Moreover, there is no effective treatment regimen for LM from lung adenocarcinoma due to lacking understanding of the molecular mechanism of LM.

MicroRNAs (miRNAs) are a group of noncoding RNAs, which can degrade messenger RNA (mRNA) and inhibit protein translation based on complementation with sequence of the target mRNAs (8). Through this approach, the altered expression of specific miRNAs is associated with cell proliferation, apoptosis, differentiation, and other biological processes (9). Moreover, it was known that some miRNAs were related to the occurrence and development of major diseases including nervous system diseases and cancers (10, 11).

CSF is a readily reachable body fluid and the most informative biofluid regarding changes that occur in the central nervous system (CNS) (12). The profiling of CSF miRNA expression indicated that specific miRNAs were related to CNS malignancies. It offers a powerful approach to understanding the development of CNS malignancies from the earliest manifestations to the terminal stages, and also provides insights about biology genetic changes of disease that might help to detect and monitor disease, as well as to explore the molecular features of the underlying malignancy (13). Over the past decades, miRNAs have been discovered to be involved in cancer metastasis (14). However, researches exploring LM-related miRNAs based on large-scale human CSF samples are rarely seen.

In this study, we first screened specific miRNAs by comprehensive miRNA microarray analyses in CSF samples from LM patients and non-LM controls, as well as in matched CSF samples from LM patients at diagnosis and after LM-directed therapy. Using quantitative real-time PCR (qRT-PCR), we then validated the expression of candidate miRNAs in CSF samples from independent LM patients with lung adenocarcinoma, and evaluated their diagnostic performance for discriminating LM patients from non-LM subjects. Next, we evaluated the effect of candidate miRNAs on proliferation, migration, and invasion of lung adenocarcinoma cell lines. The online analysis tools including TargetScan, miRDB, and miRTarbase were used to predict potential target genes of the candidate miRNA. Gene ontology (GO) and Kyoto Encyclopedia of Genes and Genomes (KEGG) were used to analyze the functions of predicted target genes. The present study aimed to investigate LM-related miRNAs and their potential value in disease diagnosis and monitoring, and to explore the possible molecular mechanisms based on experimental validation and bioinformatic analysis.

## Methods

### Patients, CSF Samples, and Study Design

In total, we collected 186 CSF samples from three groups of participants: LM patients with lung adenocarcinoma, brain metastasis patients with lung adenocarcinoma, and non-cancer controls at the First Hospital of Jilin University, China, between September 2014 and December 2018. All samples were distributed to discovery and validation sets. Detailed clinical data are summarized in Tables 1, 2.

**Table 1:** Characteristics of LM patients with lung adenocarcinoma and non-LM controls of all sets.

	**Discovery set** **(*N* = 20)**	**Validation set** **(*N* = 116)**
**Categories**		
LMs with lung adenocarcinoma	10	68
**Non-LMs**		
BMs with lung adenocarcinoma	5	43
Non-cancers	5 (2 cesarean sections and 3 appendicitis)	5 (1 cesarean section and 4 appendicitis)
**Gender**		
Female	13	66
Male	7	50
Age median (range)	52 years (28-73)	54 years (30-70)
**Neurological symptoms and signs**		
Yes	9	87
No	11	29
**CSF cytology**		
Positive	10	56
Negative	10	60
**Neuroimaging (MRI)**		
Positive for LM	9	49
No evidence of LM	6	62
Not performed	5	5

*LM, leptomeningeal metastasis; BM, brain metastasis; CSF, cerebrospinal fluid; MRI, magnetic resonance imaging*.

**Table 2:** Characteristics of six LM patients enrolled for paired CSF analyses in discovery set.

**Patient no**.	**Age (year)**	**Gender**	**Neurological symptoms and signs**	**CSF cytology**	**MRI abnormalities**	**Systemic treatment for primary tumor before LM**	**LM-directed treatment**
I	46	Female	Headache	Positive	Nodular/linear enhancement	Chemotherapy	WBRT with concurrent intrathecal MTX
II	37	Male	Dizziness, headache, nausea, vomiting	Positive	Nodular/linear enhancement	Chemotherapy	WBRT with concurrent intrathecal Ara-c
III	62	Female	Dizziness, hearing loss, tinnitus	Positive	Nodular enhancement	Icotinib	WBRT with concurrent intrathecal MTX
IV	49	Female	Headache, nausea, visual disturbance, seizures	Positive	Nodular enhancement	Chemotherapy Erlotinib	WBRT with concurrent intrathecal MTX
V	52	Male	Dizziness, headache, nausea, vomiting	Positive	linear enhancement	Erlotinib	WBRT with concurrent intrathecal MTX
VI	50	Female	Headache, vomiting	Positive	Nodular enhancement	Icotinib	WBRT with concurrent intrathecal Ara-c

*LM, leptomeningeal metastasis; CSF, cerebrospinal fluid; MRI, magnetic resonance imaging; MTX, methotrexate; Ara-c, cytarabine; WBRT, whole brain radiotherapy*.

In the discovery phase, we performed comprehensive miRNA microarray analysis in CSF samples from 10 LM patients with lung adenocarcinoma and 10 non-LM controls (5 brain metastases from lung adenocarcinoma and 5 non-cancers). Meanwhile, matched CSF samples collected from 6 LM patients at diagnosis and after efficacious LM-directed therapy were also analyzed by miRNA microarray. MiRNAs differentially expressed in common between the two comparative models of miRNA microarray were determined as candidate miRNAs. Subsequently, candidate miRNAs were quantified in CSF samples from an independent validation cohort including 68 LM patients and 48 non-LM controls (43 brain metastases from lung adenocarcinoma and 5 non-cancers).

To investigate if candidate miRNAs were involved in the disease course of LM, 22 matched CSF samples were collected and analyzed at diagnosis and after efficacious initial therapy, as well as 8 sequential CSF samples were collected and analyzed at four time points: at diagnosis, after efficacious initial therapy, at relapse, and after salvage therapy. Detailed clinical data are summarized in Table 3.

**Table 3:** Characteristics of LM patients with lung adenocarcinoma for serial analyses of CSF miRNAs.

**Patient no**.	**Age (year)**	**Gender**	**Neurological symptoms and signs**	**CSF cytology**	**MRI abnormalities**	**Systemic treatment for primary tumor before LM**	**Initial LM-directed treatment**	**Interval between initial LM diagnosis and relapse**	**LM salvage treatment**
1	53	Female	Headache, visual disturbance, seizures	Positive	Negative	None	WBRT with concurrent intrathecal MTX		
2^*^	39	Male	Dizziness	Positive	Nodular/Linear enhancement	Crizotinib, lorlatinib	WBRT with concurrent intrathecal Ara-c	9.6 months	Intrathecal pemetrexed
3^*^	38	Female	Dizziness, headache, nausea, vomiting	Positive	Linear enhancement	Icotinib, Osimertinib	WBRT with concurrent intrathecal MTX; intrathecal Ara-c	14.4 months	Intrathecal pemetrexed
4	60	Male	Dizziness, headache, diplopia, memory loss	Positive	Nodular/Linear enhancement	Chemotherapy Gefitinib	WBRT with concurrent intrathecal Ara-c		
5	57	Female	Cranial nerve palsies	Negative	Linear enhancement	Crizotinib	WBRT with concurrent intrathecal Ara-c		
6	50	Female	Headache, nausea, vomiting	Positive	Nodular enhancement	Chemotherapy	WBRT with concurrent intrathecal Ara-c		
7^*^	37	Female	Dizziness, headache, vomiting	Positive	Linear enhancement	Icotinib, Osimertinib	WBRT with concurrent intrathecal MTX; intrathecal Ara-c	8.2 months	Intrathecal pemetrexed
8	47	Male	Dizziness, headache	Positive	Nodular/Linear enhancement	Gefitinib	WBRT with concurrent intrathecal pemetrexed		
9	52	Female	Dizziness, headache, diplopia	Positive	Negative	Icotinib, Chemotherapy	WBRT with concurrent intrathecal pemetrexed		
10	59	Female	Dizziness, headache, diplopia, hearing loss	Positive	Linear enhancement	Gefitinib	WBRT with concurrent intrathecal pemetrexed		
11	61	Female	Conscious disturbance	Negative	Nodular/Linear enhancement	Afatinib	WBRT with concurrent intrathecal pemetrexed		
12^*^	57	Female	Conscious disturbance, headache	Positive	Linear enhancement	Chemotherapy, Icotinib, Osimertinib	WBRT with concurrent intrathecal MTX; intrathecal Ara-c	13.2 month	Intrathecal pemetrexed
13	66	Female	None	Positive	Nodular enhancement	Osimertinib	WBRT with concurrent intrathecal Ara-c		
14^*^	47	Female	Headache, seizures	Positive	Linear enhancement	Gefitinib, Icotinib, Osimertinib	WBRT with concurrent intrathecal MTX; intrathecal MTX	38.4 months	Intrathecal pemetrexed
15	61	Female	Dizziness, headache, vomiting, conscious disturbance	Positive	Nodular/Linear enhancement	None	WBRT with concurrent intrathecal Ara-c/MTX		
16	52	Male	Dizziness, headache, vomiting	Positive	Linear enhancement	Erlotinib	WBRT with concurrent intrathecal MTX		
17^*^	49	Female	Dizziness, vomiting, seizures	Positive	Nodular/Linear enhancement	Gefitinib, Icotinib, Osimertinib, Chemotherapy	WBRT with concurrent intrathecal MTX	9.8 months	Intrathecal pemetrexed
18^*^	66	Male	Dizziness, headache	Positive	Nodular/Linear enhancement	None	WBRT with concurrent intrathecal MTX; intrathecal Ara-c	3.2 months	Intrathecal pemetrexed
19	60	Male	Diplopia, headache	Positive	Nodular enhancement	None	WBRT with concurrent intrathecal Ara-c		
20	58	Male	Dizziness, headache	Positive	Nodular enhancement	Chemotherapy	WBRT with concurrent intrathecal Ara-c		
21	64	Female	Headache, sensory motor deficits of extremities	Positive	Nodular enhancement	Icotinib	WBRT with concurrent intrathecal Ara-c		
22^*^	55	Female	Headache, nausea, vomiting	Positive	Nodular/Linear enhancement	Gefitinib, Osimertinib	WBRT with concurrent intrathecal MTX	7.2 months	Intrathecal pemetrexed

* Eight LM patients presented recurrence at 3–38 months after initial LM-directed therapy and then received salvage therapy.

*LM, leptomeningeal metastasis; CSF, cerebrospinal fluid; MRI, magnetic resonance imaging; MTX, methotrexate; Ara-c, cytarabine; WBRT, whole brain radiotherapy*.

The diagnosis of lung adenocarcinoma was confirmed by histopathology. The non-cancer controls were patients without cancerous disease who underwent lumbar anesthesia prior to the necessary surgery. The diagnosis of LM for patients enrolled in this study was based on positive CSF cytology or suggestive clinical and neuroimaging findings meeting the diagnosis criteria recommended by European Association for Neuro-Oncology and European Society for Medical Oncology (EANO–ESMO) Clinical Practice Guidelines (6). Recurrence and progression were ascertained by at least three neuro-oncologists referring to the following conditions: (1) the deterioration of neurological symptoms/signs was progressive, typically associated with LM for more than 1 week; (2) increased intracranial pressure > 300 mmH_2_O and/or CSF glucose <2.3 mmol/L; (3) positive CSF cytology; (4) LM-related neuroimaging findings were worsening. Meanwhile, treatment-related side effects and other diseases that may lead to the above conditions should be excluded. The evaluation criteria for clinical response to LM-directed treatment were established on the improvement of neurologic symptoms/signs, CSF, and neuroimaging according to EANO–ESMO Clinical Practice Guidelines (6).

LM CSF samples in this study were from patients enrolled in the following clinical trials: ChiCTR-OOC-14005403, NCT03082144, NCT03101579, and NCT03507244. Written informed consents were obtained from all participants, which indicated willingness to donate their CSF samples for research. The study received approved from Institution Ethics Committee of the First Hospital of Jilin University and in accordance with the principles of the Declaration of Helsinki. Permission to access and publish patient information was obtained from each of the patients.

### CSF Collection and Preparation

The CSF samples from LM patients were collected at the time of diagnostic lumbar puncture or intrathecal chemotherapy. For brain metastasis patients with lung adenocarcinoma who were suspected of suffering LM but ultimately excluded, CSF samples were obtained by diagnostic lumbar puncture. The CSF samples of non-cancer controls were collected at the time of lumbar anesthesia prior to the necessary surgery. At least 2 ml of each CSF sample was stored in aliquots within 1 h after collection at −80°C until further processing. The blooded CSF samples were excluded.

### miRNA Expression Profiling Using Microarray Analysis

Microarray analysis was performed using Agilent Human miRNA (8^*^60K) arrays. The microarray contains probes for 2,549 human miRNAs from miRbase v21.0. The miRNA molecules were labeled using the miRNA Complete Labeling and Hyb Kit from Agilent, following the manufacturer's standard protocol. Each slide was hybridized with 100 ng Cy3-labeled RNA using miRNA Complete Labeling and Hyb Kit (Cat # 5190-0456, Agilent technologies, USA) in hybridization oven at 55°C for 20 h. After hybridization, slides were washed in staining dishes (Cat # 121, Thermo Shandon, USA) with Gene Expression Wash Buffer Kit (Cat # 5188-5327, Agilent technologies, USA). Then slides were scanned by Agilent Microarray Scanner (Cat # G2565CA, Agilent technologies, USA), and the images were processed with Feature Extraction software 10.7 (Agilent technologies, USA) with default settings.

### Cell Lines and Cell Transfection

The human lung adenocarcinoma cell lines NCI-H1650 and A549, kindly provided by the First Hospital of Jilin University (Changchun, China), were cultured in RPMI-1640 Medium (Cat # R8758, SIGMA, USA) and Dulbecco's Modified Eagle's Medium (DMEM; Cat # D6429, SIGMA, USA), respectively, supplemented with 10% fetal bovine serum (FBS; Cat # 010101, Trinity Tek, Spain), 100 U/ml penicillin, and 100 mg/ml streptomycin (Cat # P4333, SIGMA, USA) in a humidified incubator at 37°C in a 5% CO_2_ atmosphere.

Cells were seeded in six-well plates (2 × 10^5^ cells per well) overnight prior to transfection. MiRNA mimics (50 nM) and negative control (50 nM) were transfected into the cells by Lipofectamine 3000 reagent (Cat # L3000015, Invitrogen, USA) in accordance with the manufacturer's instruction. The miRNA mimics and mimic negative control were purchased from GenePharma Co., Ltd (Shanghai, China).

### RNA Extraction and Quantitative Real-Time Polymerase Chain Reaction Analysis

Total RNA from CSF samples was extracted and purified using QIAamp Circulating Nucleic Acid Kit (Cat # 55114, QIAGEN, USA), following the manufacturer's instructions and checked for an RNA integrity number to inspect RNA integration by an Agilent Bioanalyzer 2100 (Agilent technologies, USA). For lung adenocarcinoma cells, total RNA was extracted using miRcute miRNA isolation kit (Cat # DP501, Tiangen Biotech Co., Ltd, Beijing, China) at 24 h after transfection with miRNA mimics or negative control nucleotides.

Then miRNA was reverse transcribed with miScriptII RT Kit (Cat # 218161, QIAGEN, USA), and quantified by qRT-PCR using the miScriptSYBR® Green PCR Kit (Cat # 218073, QIAGEN, USA) and commercial primers (hsa-miR-7975, hsa-miR-7977, miR-7641, miR-4800-5p, QIAGEN, USA). All samples were run in triplicate on Line Gene 9600 Plus system (Bioer Techonogy, Hangzhou, China) using default settings. *C. Elegans* miR-39 (cel-miR-39, primer from QIAGEN, USA) in CSF samples and U6 (primer from Ribobio Co. Ltd, Guangzhou, China) in cell lines were used for control. The relative expression level (REL) of each miRNA in CSF samples and cell lines was calculated with the 2^−ΔCt^ and 2^−ΔΔCt^ methods, respectively.

### Cell Proliferation Assay

At 24 h after transfection with miRNA mimics and negative control, cells were seeded into 96-well plates at a density of 5 × 10^3^ per well. After incubation for another 12, 24, and 48 h, 10% Cell Counting Kit-8 (CCK-8; Cat # GK10001, GLPBIO, Montclair, CA, USA) diluted in normal culture medium was added to each well and incubated for an additional 4 h. The absorbance was measured spectrophotometrically at 450 nm. Each experiment was performed at least three times independently.

### Wound Healing Assay

The changes in cell migration ability were detected by wound healing assay. Cells were seeded in six-well plates. At 24 h after transfection with miRNA mimics or negative control, wounds were made in the cell layer using a sterile micropipette tip. Then, the cells were washed with PBS buffer. The width of the wound gap was viewed under a microscope and photographed at 0, 9, and 24 h after wounding. Three replicate wells from a six-well plate were used for this experiment.

### Cell Invasion Assay

The invasion assay was performed with a transwell chamber inserted with a Polyester (PET) filter membrane (8 μM pores, Corning, USA) in 24-well plates (Corning, USA). The top side of the filter was coated with Matrigel. At 24 h after transfection with mimics or negative controls, the cells (1 × 10^5^/ml) were collected and resuspended in 200 μl serum-free medium and added to the top well. Six hundred milliliter medium containing 15% FBS was added to the lower compartment. After 48 h of incubation, the cells on the upper surface of the membrane were carefully removed with a cotton bud. The cells that invaded through the PET filter membrane were fixed with cold methanol for 30 min, stained with 0.1% crystal violet for 20 min, then photographed and counted under a microscope.

### Prediction of Target Genes

The potential target genes of the differentiated expressed miRNAs are predicted using TargetScan, miRDB, and miRTarbase online analysis tools. The overlapping target genes were identified using Venn diagram in order to further enhance the bioinformatics analysis reliability.

### Gene Ontology and KEGG Pathway Analysis

The functional annotation and pathway enrichment analysis including GO and KEGG pathway analysis (15, 16) were performed for the predicted overlapping target genes of the differentiated expressed miRNAs using database for annotation, visualization, and integrated discovery (DAVID, https://david.ncifcrf.gov). The criteria of cut-off were gene count ≥ 3.

### Statistical Analysis

GraphPad Prism (version 7.0, GraphPad Software) and SPSS (version 22.0, SPSS Software) were used for all statistical analyses. The data were expressed as mean ± standard deviation (SD). Differential expression of miRNAs was analyzed by the unpaired *T*-test, Mann–Whitney test, and Wilcoxon matched-pairs signed rank test. A value of *P* < 0.05 was regarded as statistically significant. Receiver operating characteristic (ROC) curve analysis was used to evaluate diagnostic performance.

## Results

### Profiling of CSF miRNAs in Leptomeningeal Metastasis Patients

**Figure 1:**
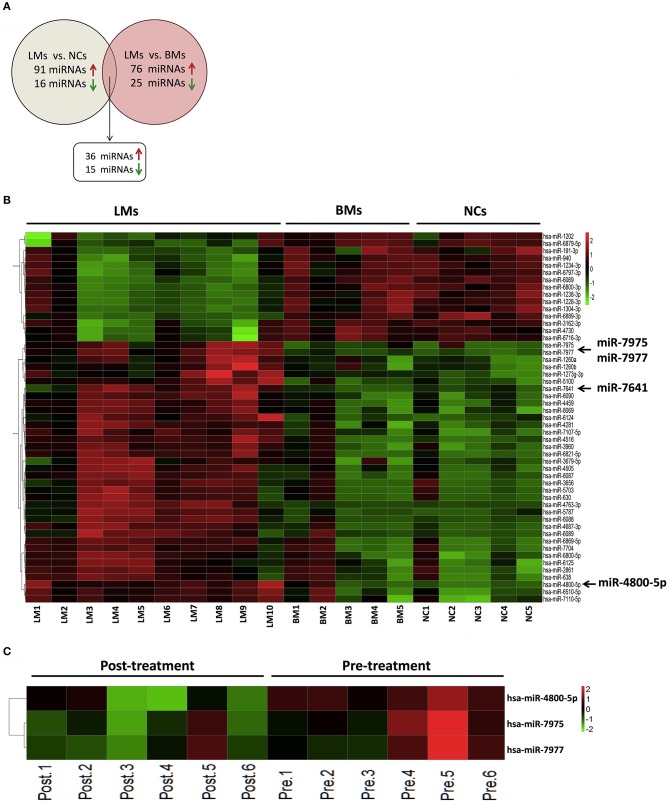
Differential expression of miRNAs in CSF from discover cohort. **(A)** The overlapping miRNAs differentially expressed between two comparison models (LMs, *n* = 10; BMs, *n* = 5; NCs, *n* = 5); **(B)** Heatmap of differentially expressed CSF miRNAs analyzed by microarray between LMs (*n* = 10) and non-LMs controls (BMs, *n* = 5; NCs, *n* = 5); **(C)** Heatmap of differentially expressed miRNAs analyzed by microarray in paired CSF samples from 6 LM patients at diagnosis and after initial LM-directed therapy. Fold change ≥2 or ≤0.5 was regarded as statistically significant. CSF, cerebrospinal fluid; LMs, leptomeningeal metastases; BMs, brain metastases; NCs, non-cancers.

To evaluate whether specific miRNA signatures were detectable in CSF samples from LM patients with lung adenocarcinoma, a microarray analysis of 2,549 human miRNAs was performed. The criterion of each mappable miRNA was chosen for further analysis based on differentially expressed gene analysis with fold change values of more than 2 and < 0.5. Based on the criteria described, 36 miRNAs upregulated and 15 miRNAs downregulated consistently in LM when compared with brain metastasis and non-cancer controls, respectively, were identified for further analysis (Figures 1A,B and Supplemental Table 1). The raw miRNA data are available from the NCBI Gene Expression Omnibus (GEO) through series accession number GSE125193.

In order to screen specific miRNAs correlated with the development of LM, we also performed miRNA microarray in CSF samples of six LM patients at diagnosis and their matched CSF samples after efficacious LM-directed therapy. Compared with the levels at diagnosis, CSF miR-7975, miR-7977, and miR-4800-5p were significantly decreased after efficacious therapy (Figure 1C and Supplemental Table 2). The raw miRNA data are available from the NCBI GEO through series accession number GSE125193. As we have confirmed that the three miRNAs were significantly upregulated in LMs compared with non-LMs, the results indicate that the three miRNAs may be involved with the development of LM and, therefore, were selected for further validation. In addition, CSF miR-7641 with the largest fold-change value of 58.3 in LMs vs. non-LMs was also selected as a candidate miRNA.

### Validation of Candidate CSF miRNAs and Evaluation of Their Diagnostic Performance

**Figure 2:**
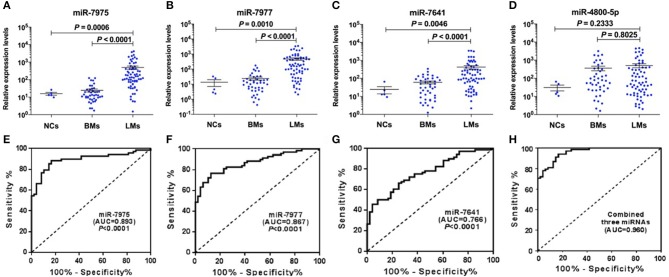
Validation of candidate CSF miRNAs and evaluation of their diagnostic performance in validation cohort. RELs of CSF miR-7975 **(A)**, miR-7977 **(B)**, miR-7641 **(C)**, and miR-4800-5p **(D)** in 68 LM patients vs. 48 non-LM controls (BMs, *n* = 43; NCs, *n* = 5) were detected by qRT-PCR. Then ROC curve analysis with RELs of CSF miRNAs including miR-7975 **(E)**, miR-7977 **(F)**, miR-7641 **(G)**, and the combined three CSF miRNAs **(H)**, was performed for discrimination of LMs and non-LM controls. RELs of miRNAs (y-axis) are normalized to cel-miR-39. The black horizontal lines represent median REL values with SEM. A value of *P* < 0.05 was regarded as statistically significant. RELs, relative expression levels; LMs, leptomeningeal metastases; BMs, brain metastases; NCs, non-cancers; SEM, standard error of mean; AUC, area under curve.

The robustness of the miRNA microarray data was studied by analyzing their levels using qRT-PCR in an independent validation set, which includes CSF samples from an additional 68 LM patients with lung adenocarcinoma and 48 non-LM controls. MiRNA expression data were normalized using cel-miR-39 levels in individual CSF specimens and reported as RELs. In concordance with the results presented in the discovery set, we found a significant difference for miR-7975, miR-7977, and miR-7641 between CSF samples from LM patients and non-LM controls (Figures 2A–C). However, for miR-4800-5p, we didn't observe the consistent result with microarray analysis (Figure 2D).

To demonstrate the utility of candidate CSF miRNAs in discriminating cases of LM with lung adenocarcinoma from non-LM subjects, ROC curve analysis was performed among the validation set. The results showed that the area under curve (AUC) value for miR-7975, miR-7977, and miR-7641 were 0.893 (*P* < 0.0001, 95% CI 0.832–0.953), 0.867 (*P* < 0.0001, 95% CI 0.803–0.931), and 0.766 (*P* < 0.0001, 95% CI 0.682–0.850), respectively (Figures 2E–G). The diagnostic sensitivity and specificity were 88.24 and 83.33% for miR-7975, 76.47, and 87.50% for miR-7977, and 50.00% and 91.67% for miR-7641. Compared with miR-7641, both CSF miR-7975 and miR-7977 with AUC values of more than 0.85 exhibited better diagnostic performance in delineating LMs from non-LM subjects. In addition, the combined three miRNAs exhibited the best performance in distinguishing LM cases from non-LM subjects with an AUC value of 0.960 (95% CI 0.930–0.989), 94.10% sensitivity, and 83.33% specificity (Figure 2H). These data strongly indicated that CSF miRNAs may become reliable diagnostic markers for LM with lung adenocarcinoma.

### Identification of Candidate CSF miRNAs Expression Through the Course of Leptomeningeal Metastasis

**Figure 3:**
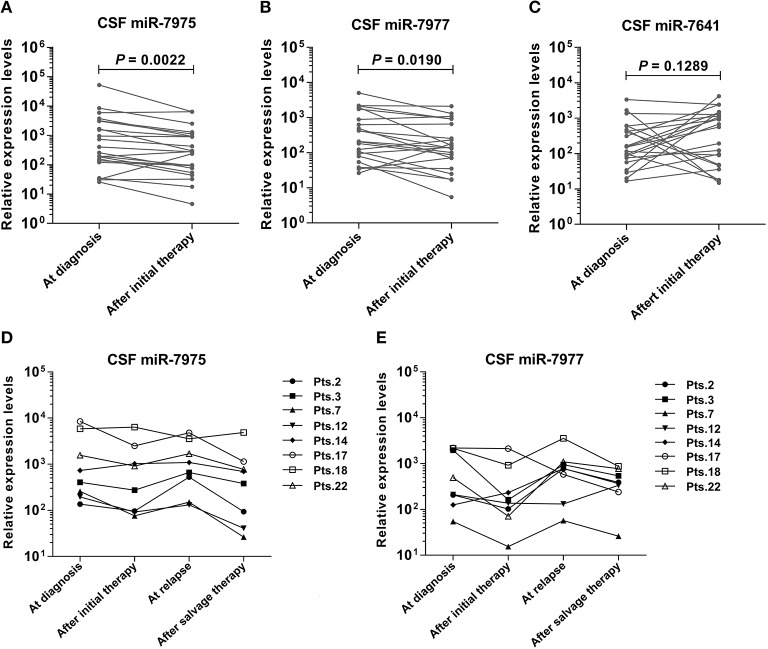
Identification of specific CSF miRNAs expression through the course of LM. RELs of miRNA were compared between matched CSF samples from 22 LM patients at diagnosis and after initial LM-directed therapy. Compared with the levels at diagnosis, CSF miR-7975 **(A)** and miR-7977 **(B)** were significantly down regulated in LM patients after initial efficacious therapy, while CSF miR-7641 **(C)** was not. Then miR-7975 and miR-7977 in sequential CSF samples from 8 LM patients collected at four time points: at diagnosis, after initial therapy, at relapse and after salvage therapy were analyzed. Longitudinal REL data of both CSF miR-7975 **(D)** and miR-7977 **(E)** in 6 out of 8 LM patients correlated well with the clinical courses of disease, decreasing after initial therapy, rising during relapse, and again returning to lower levels after salvage therapy. Lines, matched samples. RELs of miRNAs (y-axis) are normalized to cel-miR-39. A value of *P* < 0.05 was regarded as statistically significant. RELs, relative expression levels; LM, leptomeningeal metastasis.

To observe the relationship between candidate CSF miRNAs expression and disease course, longitudinal studies were performed. RELs of the three miRNAs (miR-7975, miR-7977, and miR-7641) were compared in CSF samples of 22 LM patients at initial diagnosis with their matched CSF samples after efficacious LM-directed therapy. Compared with the levels at diagnosis, CSF miR-7975 and miR-7977 were significantly down-regulated in LM patients after initial efficacious therapy (Figures 3A,B), while CSF miR-7641 was not (Figure 3C). To further investigate if levels of specific CSF miRNAs could be used to monitor the disease course, sequential CSF samples from eight LM patients were collected at four time points: at diagnosis, after initial therapy, at relapse, and after salvage therapy. Strikingly, longitudinal REL data of both CSF miR-7975 and miR-7977 in six out of eight LM patients correlated well with the clinical courses of LM (Figures 3D,E), decreasing after initial therapy, rising during relapse, and returning to lower levels after salvage LM-directed therapy. Although these observations are based on individual cases, these data support the application value of CSF miRNA levels for monitoring of disease course in LM.

### *In vitro* Effects of miR-7975 and miR-7977 on Proliferation, Migration, and Invasion of Lung Adenocarcinoma Cells

**Figure 4:**
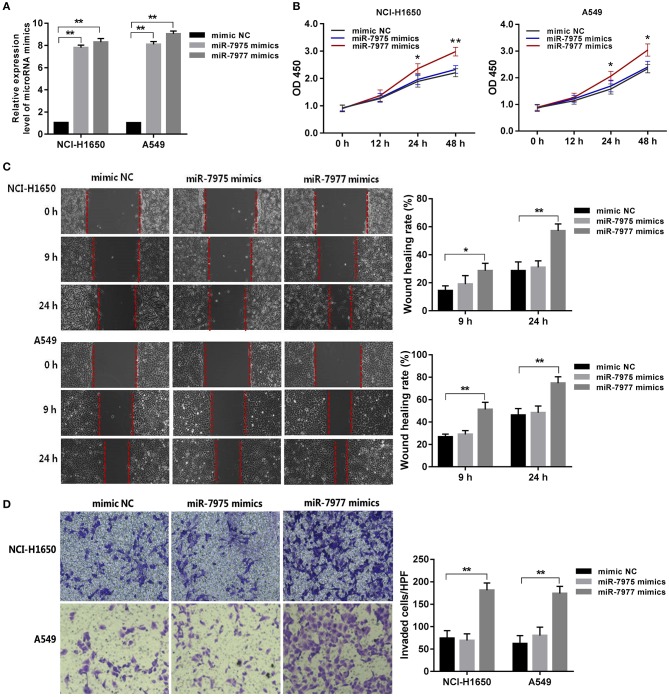
The role of miR-7977 in regulating lung adenocarcinoma cell proliferation, migration, and invasion. After transfection with miRNA mimics and negative control at final concentrations of 50 nM for 24 h: **(A)** the expression levels of miR-7975 and miR-7977 in NCI-H1650 and A549 cells were detected using qRT-PCR; **(B)** the proliferation assay of NCI-H1650 and A549 cells was performed using CCK-8 kit at three time points: 12, 24, and 48 h; **(C)** wounds were made in NCI-H1650 and A549 cells layer using a sterile micropipette tip, and the width of the wound gap was viewed under a microscope and photographed at 0, 9, and 24 h after wounding; **(D)** the invasion assay of NCI-H1650 and A549 cells was performed using Matrigel-coated transwell chambers at another 48 h of incubation. The data were expressed as mean ± SD. Each experiment was performed at least three times independently on different days. *P < 0.05, **P < 0.0001. mimic NC, mimic negative control; SD, standard deviation.

In the first place, the expression levels of miR-7975 and miR-7977 in NCI-H1650 and A549 cells after transfected with miRNA mimics at final concentrations of 50 nM were detected using qRT-PCR (Figure 4A). To assess the effect of miR-7975 and miR-7977 on the proliferation of NCI-H1650 and A549 cells *in vitro*, we transfected the cells with 50 nM miRNA mimics or negative control nucleotides. The CCK-8 assay showed that overexpressing miR-7977 increased the proliferation of NCI-H1650 and A549 cells at 24 and 48 h, while transfection with miR-7975 did not affect cell proliferation (Figure 4B). Then, a wound healing assay was performed to determine the roles of miR-7975 and miR-7977 in cells migration. As shown in Figure 4C, images of cell migration were obtained at the edge of the scratch at 0, 9, and 24 h. After 24 h, the wound gap was markedly closer in the miR-7977 mimic groups than in the negative control groups. The results suggested that miR-7977 promoted the motility of NCI-H1650 and A549 cells *in vitro*. However, overexpression of miR-7975 could not significantly affect cell migration, when compared with negative control cells. Next, we investigated the effect of miR-7975 and miR-7977 on the invasion of lung adenocarcinoma cells by using Matrigel-coated transwell chambers. The results showed that the numbers of cells that crossed the Matrigel-coated filter membrane were significantly increased in the miR-7977 mimic groups compared to those in negative control groups after 48 h (Figure 4D), while upregulation of miR-7975 did not affect cellular invasion. All these data confirmed that miR-7977 was a key regulator of proliferation, migration, and invasion in lung adenocarcinoma.

### Target Gene Prediction and Function Analysis of miR-7977

**Figure 5:**
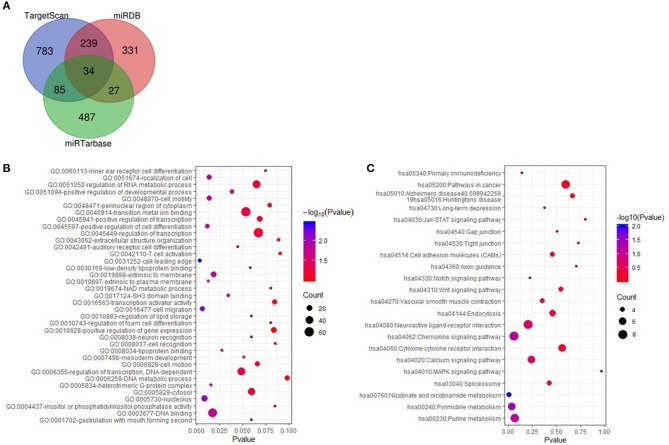
The targeted genes of miR-7977 prediction and function anlysis. **(A)** The overlapping targeted genes were predicted using TargetScan, miRDB and miRTarbase online analysis tools; **(B)** The enriched GO annotation for target genes of miR-7977 including biological process, cellular component and molecular function; **(C)** The enriched KEGG pathways for target genes of miR-7977. The gene count ≥ 3 were set as the cut-off criteria.

The target genes of miR-7977 were predicted using TargetScan, miRDB, and miRTarbase online analysis tools. A total of 385 overlapping genes in any two databases was identified (Figure 5A). Then, GO function annotation analyses including biological process, cellular component, and molecular function were performed on these potential target genes (Figure 5B). It was worth noting that the GO biological process terms were mainly enriched in cell migration, regulation of cell migration, and regulation of cell differentiation, and localization of cell. Subsequently, we conducted KEGG pathway enrichment analysis to further analyze the enriched pathways of these target genes. As shown in Figure 5C, the enriched KEGG pathways included chemokine signaling pathway, cell adhesion molecules, gap junction, Wnt signaling pathway, pathways in cancer, tight junction, Jak-STAT signaling pathway, and mitogen-activated protein kinase (MAPK) signaling pathway. In all the three databases, there were four overlapping target genes including CCL22, STK4, HSPA1B, and ACTR2, which were associated with cell migration, chemokine signaling pathway, and MAPK signaling pathway.

## Discussion

LM is a typically late complication of solid tumors. Diagnosis and monitoring of LM are challenging. Additionally, it implies a poor prognosis and limited treatment options (3). Therefore, study biomarkers and mechanisms of development in LM are of great significance for improving the prognosis of LM and provide a new target for design of good therapy. In the present study, we systematically investigated the miRNA profiles of a relatively large number of CSF samples from LM patients with lung adenocarcinoma and non-LM patients. It demonstrated for the first time that three CSF miRNA (miR-7975, miR-7977, and miR-7641) levels were higher in lung adenocarcinoma patients with LM than those without LM. The combined three miRNAs exhibited optimal diagnostic performance, suggesting that they may be potential diagnostic biomarkers for LM. Furthermore, the dynamic change in expression level of these CSF miRNAs at diagnosis, after initial therapy, at relapse, and after salvage therapy showed that the expression levels of CSF miR-7975 and miR-7977 were concordant with the course of disease, which indicated the application value of the two miRNAs for monitoring LM course. In addition, the results of both experiments on cell biological functions and bioinformatics analysis suggested that miR-7977 is a key factor for LM in lung adenocarcinoma, which develops a new way for exploring the mechanism of LM.

It is essential to make early diagnosis for efficacious treatment and improvement of disease prognosis, because LM represents a devastating cancer complication. Several researches on LM have evaluated the diagnostic accuracy of CSF cytology and magnetic resonance imaging (MRI) methods (17, 18). However, both of these methods are highly examiner-dependent and have limitations of inter-reader variability and non-specific findings, which prompted studies evaluating other CSF biomarkers (19–23). The findings of our study were that CSF miR-7975, miR-7977, and miR-7641 could discriminate LM patients from non-LM subjects. It was worth noting that the combination of the three CSF miRNAs had a significant diagnostic value for LM and yielded an AUC of 0.960 in ROC analysis. The data of this study strongly indicates that the analysis of miRNA in CSF is a promising approach for exploring minimally invasive screening examinations for LM. Since repeated lumbar puncture for CSF cytology examination is unacceptable for brain metastasis patients with suspected LM, these findings are of great importance, especially to differentiate LM patients from brain metastasis patients at the time of initial CSF examination.

LM patients frequently suffer from disease relapse or progression. So far, there is still no definite biomarker for reflecting disease course of LM. Generally, if the development of the disease during different stages has the same pathologic process, ideal diagnostic biomarkers can be used for disease monitoring (24). A previous report has suggested using miRNA detection in CSF to monitor the course of primary CNS lymphoma (25). In this study, we addressed CSF miRNA levels over time of individual LM patients as potential markers for the purpose of monitoring disease course. In our pilot study, longitudinal detections of CSF miRNA levels in LM patients were performed, the results of which indicate that the differential expression of CSF miR-7975 and miR-7977 was correlated with the LM disease course. Although observations here are based on a small number of cases, our study provides the rationale for future investigations of CSF miRNAs as biomarkers for monitoring disease course of LM patients in larger cohorts.

The identification of specific CSF miRNAs in this study not only provides a new method for diagnosing and monitoring LM but also develops a new way for exploring the mechanism of LM. The molecular mechanism of LM is still elusive, which may be the main obstacle to seeking efficacious treatment. Currently, efforts to better understand the molecular mechanism of LM have been severely limited by the difficulties in isolating tumor cells from CSF as well as characterizing them. Increasing evidence emerging from preclinical models of different tumor types clearly indicates that specific miRNAs play a functional role in different steps of the metastatic cascade (26). In our study, CSF miR-7975 and miR-7977 are up-regulated specifically in patients of LM from lung adenocarcinoma, but not in those of brain metastases from lung adenocarcinoma and non-cancers. Moreover, both of them have been confirmed to be closely related with the course of LM disease. Although the biologic significance of these specific CSF miRNAs in LM patients needs to be established, one of them has already been associated with malignant diseases. MiR-7977 in extracellular vesicles has been demonstrated to induce failure of normal hematopoiesis via its target gene poly(rC) binding protein 1 (PCBP1) in hematologic malignancies (27). In this study, we found that overexpression of miR-7977 promoted proliferation, invasion, and migration of lung adenocarcinoma cells. Furthermore, GO function analyses showed that the targeted genes of miR-7977 were mainly involved in regulation of cell migration and cell differentiation. Based on the existing literature and our experimental results, we speculate that miR-7977 may have a function of oncogene in LM. The increase of miR-7977 expression may trigger the activation of proliferation, invasion, and migration, which were related cancer metastasis mechanisms, and promote the occurrence of LM. It provides a theoretical basis for revealing the possible mechanism of LM.

In addition, we found that the KEGG pathways of miR-7977 targeted genes included tight junction, gap junction, and other abnormal signaling pathways including the Wnt signaling pathway, Jak-STAT signaling pathway, and MAPK signaling pathway. One of the main patterns of LM is believed to occur via hematogenous route, implying that cancer cells reach the brain barrier and invade the space of CSF flow. Tight junctions have become a key factor in the studies on integrity of brain barrier including blood–brain barrier (BBB) and blood–cerebrospinal fluid barrier (BCSFB) during CNS metastasis. A previous study has confirmed that disruption of the tight junctions of the BBB is an important link in the development of brain metastasis (28, 29). Since BCSFB plays a vital role during the occurrence of LM, we speculate that the overexpression of miR-7977 may allow cancer cells to transmigrate through the BCSFB by regulating tight junctions. In addition, the four overlapping target genes of miR-7977 (CCL22, STK4, HSPA1B, and ACTR2) in all three databases were reported to have a key role in cancer formation and metastasis. Studies have shown that these genes are involved in the growth, invasion, and metastasis of a variety of tumors, and that they play a key role in tumor-related molecular mechanisms (30–33). Furthermore, abnormal signaling pathways also play crucial roles in the development of cancer metastasis. Accumulating evidence has demonstrated that activation of MAPK signaling pathway, Jak-STAT signaling pathway, and Wnt signaling pathway is important in cancer progression (34–36). Therefore, it is needed to perform further molecular investigations to confirm these predictions, and it can provide a new perspective in exploring the mechanism of LM and a new intervention method in LM treatment.

There are several limitations of this study. First, the CSF sample size was not large enough in the microarray analysis. Second, our study indicated that the specific CSF miRNAs have potential value in diagnosis and monitoring of LM. However, all the patients enrolled in this study were from our hospital. Potential drawbacks such as selection bias may occur. Further validation of the identified miRNAs in a larger independent population from multiple cancer centers is necessary before they can be put in a clinical application. Third, only experiments on biological functions *in vitro* and bioinformatics analysis of miR-7977 were performed in this study. Research on detailed molecular mechanisms that miR-7977 promotes the occurrence of LM through regulating targeted genes is lacking. Therefore, it is required to perform future investigations with larger clinical samples and corresponding experiments.

## Conclusions

In conclusion, we have successfully identified three LM-associated miRNAs (miR-7975, miR-7977, and miR-7641) based on microarray screening and qRT-PCR validation. Moreover, this study, if confirmed in prospective clinical trials, indicates that these miRNA signatures could be a significant tool to diagnose and monitor LM. In addition, the experiments *in vitro* and bioinformatic analysis suggested that miR-7977 plays a key role in the development of LM, which reveals a novel pathway to exploration of underlying mechanisms of LM, and provides a promising target in treating LM patients. CSF miRNAs are potentially of high worth for LM-related studies; however, much work still needs to be done to shepherd findings from scientific research into advanced clinical applications.

## Data Availability Statement

The datasets generated for this study can be found in tNCBI Gene Expression Omnibus (GEO) through accession number GSE125193.

## Ethics Statement

This study was approved by the Institutional Ethics Review Board of the First Hospital of Jilin University, Changchun, China, and was performed in accordance with the Declaration of Helsinki.

## Author Contributions

ZP, GY, GZ, and YC designed the study. ZP and GY wrote the manuscript. ZP, GY, TJ, and HH performed the experiments and statistical analysis of the data. TJ and PG collected clinical samples and clinical data of patients. All authors read and approved the final manuscript.

### Conflict of Interest

The authors declare that the research was conducted in the absence of any commercial or financial relationships that could be construed as a potential conflict of interest.

## Abbreviations

LM: leptomeningeal metastasis
CSF: cerebrospinal fluid
miRNAs: microRNAs
CNS: central nervous system
GO: gene ontology
KEGG: Kyoto Encyclopedia of Genes and Genomes
EANO–ESMO: European Association for Neuro-Oncology and European Society for Medical Oncology
GEO: Gene Expression Omnibus
AUC: area under curve
ROC: Receiver Operating Characteristic
MRI: Magnetic Resonance Imaging
PCBP1: poly(rC) binding protein 1
BBB: blood–brain barrier
BCSFB: blood–cerebrospinal fluid barrier.
