# Supplementary material for: Identification of Cerebrospinal Fluid MicroRNAs Associated With Leptomeningeal Metastasis From Lung Adenocarcinoma
Source: Front Oncol. 2020 Apr 3;10:387. doi: 10.3389/fonc.2020.00387 (PMC7152668; doi:10.3389/fonc.2020.00387)
Supplement: Supplementary file 1 [file Table_1.DOC]

**Supplemental Tables**

**Supplemental Table 1 The mappable miRNAs deregulated consistently in LMs** compared with brain metastases and non-cancer controls

| **Systematic**  **Name** | ***P* value*** | **Fold Change** | **LM 1** | **LM 2** | **LM 3** | **LM 4** | **LM 5** | **LM 6** | **LM 7** | **LM 8** | **LM 9** | **LM 10** | **BM 1** | **BM 2** | **BM 3** | **BM 4** | **BM 5** | **NC 1** | **NC 2** | **NC 3** | **NC 4** | **NC 5** |
| --- | --- | --- | --- | --- | --- | --- | --- | --- | --- | --- | --- | --- | --- | --- | --- | --- | --- | --- | --- | --- | --- | --- |
| hsa-miR-1202 | 5.52E-03 | 0.44 | 6.75 | 6.91 | 5.34 | 5.60 | 5.43 | 4.36 | 4.68 | 5.08 | 4.31 | 2.63 | 6.43 | 7.20 | 7.03 | 7.09 | 6.18 | 4.74 | 6.91 | 7.10 | 6.36 | 6.91 |
| hsa-miR-1228-3p | 9.82E-05 | 0.33 | 4.39 | 3.80 | 2.69 | 2.80 | 3.29 | 3.01 | 2.58 | 2.94 | 2.59 | 5.34 | 4.08 | 5.57 | 4.33 | 6.08 | 5.64 | 5.12 | 5.75 | 5.09 | 4.74 | 5.03 |
| hsa-miR-1234-3p | 4.19E-04 | 0.36 | 5.07 | 5.01 | 3.17 | 3.47 | 4.53 | 3.60 | 3.10 | 3.99 | 2.94 | 5.96 | 4.67 | 6.23 | 5.16 | 6.14 | 6.49 | 6.31 | 6.12 | 5.02 | 5.79 | 5.88 |
| hsa-miR-1238-3p | 6.82E-05 | 0.36 | 3.79 | 3.21 | 2.37 | 2.31 | 2.85 | 2.61 | 2.32 | 2.64 | 2.27 | 4.58 | 4.05 | 4.63 | 3.46 | 5.25 | 4.52 | 4.94 | 5.29 | 4.26 | 3.92 | 4.54 |
| hsa-miR-1260a | 1.02E-03 | 4.02 | 4.80 | 6.19 | 7.84 | 7.67 | 5.14 | 4.55 | 6.59 | 6.22 | 6.32 | 5.06 | 4.57 | 4.55 | 5.68 | 2.84 | 4.84 | 4.35 | 2.98 | 4.31 | 4.74 | 3.08 |
| hsa-miR-1260b | 2.09E-03 | 3.04 | 3.88 | 5.21 | 6.83 | 5.80 | 4.45 | 3.48 | 4.25 | 4.72 | 4.32 | 4.44 | 3.42 | 3.31 | 4.84 | 2.01 | 3.88 | 3.54 | 2.26 | 3.42 | 3.57 | 2.47 |
| hsa-miR-1273g-3p | 1.22E-03 | 3.23 | 4.50 | 6.62 | 5.87 | 7.19 | 4.87 | 4.28 | 4.72 | 6.37 | 4.86 | 5.76 | 4.57 | 4.39 | 4.28 | 4.28 | 4.66 | 4.24 | 3.26 | 3.93 | 3.74 | 3.27 |
| hsa-miR-1304-3p | 1.82E-04 | 0.38 | 3.59 | 3.19 | 2.31 | 2.10 | 2.66 | 2.58 | 2.27 | 2.44 | 2.25 | 4.63 | 3.57 | 4.59 | 3.41 | 5.22 | 4.74 | 4.15 | 5.14 | 3.72 | 4.14 | 4.56 |
| hsa-miR-191-3p | 2.54E-03 | 0.41 | 3.36 | 3.30 | 2.38 | 2.10 | 2.60 | 2.54 | 2.16 | 2.40 | 2.14 | 4.07 | 3.56 | 4.85 | 2.31 | 4.46 | 4.57 | 3.96 | 4.98 | 2.83 | 3.48 | 4.38 |
| hsa-miR-2861 | 1.37E-02 | 2.44 | 6.52 | 3.92 | 5.24 | 5.83 | 6.38 | 7.84 | 7.47 | 5.75 | 7.76 | 6.91 | 7.12 | 4.20 | 4.50 | 2.50 | 6.78 | 6.88 | 1.92 | 2.62 | 2.67 | 3.85 |
| hsa-miR-3162-3p | 3.18E-03 | 0.45 | 5.89 | 6.29 | 3.36 | 4.24 | 5.85 | 4.10 | 3.74 | 4.49 | 3.35 | 6.40 | 5.24 | 6.62 | 6.77 | 6.07 | 6.73 | 6.64 | 6.38 | 5.98 | 6.42 | 6.10 |
| hsa-miR-3656 | 2.16E-03 | 2.99 | 3.32 | 2.78 | 4.93 | 5.20 | 4.15 | 5.33 | 5.09 | 5.09 | 5.34 | 2.67 | 4.41 | 1.94 | 1.94 | 1.87 | 3.34 | 4.78 | 1.86 | 1.93 | 1.83 | 2.26 |
| hsa-miR-3679-5p | 9.29E-03 | 3.76 | 4.55 | 5.35 | 5.63 | 6.05 | 4.44 | 7.61 | 7.43 | 5.32 | 7.22 | 2.99 | 5.22 | 5.90 | 2.02 | 1.94 | 4.84 | 4.83 | 2.90 | 3.33 | 3.00 | 4.06 |
| hsa-miR-3960 | 1.05E-04 | 5.72 | 6.58 | 7.14 | 8.76 | 6.93 | 7.11 | 6.74 | 8.53 | 7.22 | 8.57 | 6.52 | 7.02 | 3.40 | 3.90 | 2.52 | 5.63 | 6.60 | 3.36 | 3.26 | 2.42 | 4.48 |
| hsa-miR-4281 | 2.56E-05 | 6.76 | 5.65 | 6.97 | 7.12 | 6.60 | 7.01 | 7.87 | 8.54 | 6.94 | 8.75 | 6.39 | 6.47 | 5.49 | 2.85 | 2.15 | 4.43 | 5.05 | 4.03 | 3.59 | 2.91 | 4.81 |
| hsa-miR-4459 | 1.99E-05 | 10.65 | 6.58 | 6.66 | 9.38 | 8.77 | 7.13 | 9.08 | 9.62 | 7.40 | 9.35 | 7.06 | 7.04 | 3.30 | 3.23 | 2.29 | 5.46 | 6.29 | 3.23 | 3.14 | 2.84 | 5.52 |
| hsa-miR-4505 | 1.94E-04 | 6.43 | 4.24 | 3.11 | 6.33 | 5.47 | 4.68 | 6.85 | 6.82 | 5.47 | 6.68 | 3.81 | 4.39 | 3.77 | 1.84 | 1.85 | 3.21 | 4.03 | 1.80 | 1.98 | 1.97 | 3.24 |
| hsa-miR-4516 | 1.80E-06 | 11.46 | 5.72 | 7.38 | 9.25 | 6.38 | 6.78 | 6.34 | 7.15 | 6.76 | 7.55 | 6.09 | 6.20 | 2.02 | 2.10 | 1.88 | 4.39 | 3.49 | 3.12 | 2.01 | 1.77 | 2.65 |
| hsa-miR-4687-3p | 1.20E-05 | 10.22 | 4.81 | 3.21 | 5.49 | 6.40 | 6.53 | 6.54 | 7.95 | 6.54 | 7.97 | 6.22 | 5.73 | 1.93 | 1.86 | 1.83 | 3.26 | 3.29 | 1.86 | 1.96 | 1.80 | 2.45 |
| hsa-miR-4730 | 1.63E-03 | 0.38 | 5.69 | 6.21 | 2.37 | 4.36 | 5.43 | 3.82 | 3.81 | 4.57 | 3.02 | 5.40 | 5.61 | 6.74 | 7.17 | 5.86 | 6.59 | 5.88 | 5.39 | 6.62 | 6.36 | 5.60 |
| hsa-miR-4763-3p | 2.33E-04 | 10.18 | 2.96 | 2.25 | 4.31 | 5.92 | 5.20 | 5.64 | 6.17 | 5.89 | 6.07 | 2.75 | 2.48 | 1.75 | 1.88 | 1.75 | 1.86 | 2.00 | 1.79 | 1.87 | 1.79 | 1.86 |
| hsa-miR-4800-5p | 2.11E-05 | 3.42 | 3.64 | 5.51 | 4.51 | 3.89 | 4.05 | 3.70 | 3.95 | 4.46 | 3.86 | 5.24 | 4.24 | 2.30 | 2.09 | 1.90 | 3.41 | 2.32 | 2.01 | 1.74 | 1.78 | 2.19 |
| hsa-miR-5100 | 1.58E-05 | 2.98 | 3.84 | 5.56 | 4.84 | 5.53 | 4.21 | 3.83 | 4.27 | 4.75 | 4.31 | 4.05 | 4.10 | 2.95 | 2.54 | 2.93 | 2.68 | 3.62 | 2.26 | 3.02 | 2.80 | 2.92 |
| hsa-miR-5703 | 1.94E-03 | 6.25 | 3.83 | 2.57 | 6.16 | 6.39 | 5.47 | 7.03 | 8.42 | 6.21 | 7.56 | 4.10 | 5.54 | 1.85 | 1.73 | 1.65 | 4.61 | 5.88 | 1.72 | 1.72 | 1.76 | 2.57 |
| hsa-miR-5787 | 3.84E-05 | 4.41 | 3.03 | 3.07 | 4.39 | 4.63 | 4.20 | 5.09 | 5.11 | 4.51 | 5.28 | 2.87 | 3.13 | 2.14 | 1.96 | 2.19 | 2.16 | 1.96 | 2.77 | 1.92 | 1.92 | 2.35 |
| hsa-miR-6069 | 2.02E-05 | 0.29 | 4.25 | 3.78 | 2.62 | 2.89 | 3.52 | 2.71 | 2.56 | 2.87 | 2.50 | 5.15 | 4.30 | 5.49 | 5.21 | 5.94 | 5.27 | 5.18 | 5.56 | 5.71 | 4.97 | 5.20 |
| hsa-miR-6087 | 4.42E-05 | 14.66 | 6.42 | 4.08 | 8.11 | 7.93 | 7.30 | 9.95 | 9.47 | 7.47 | 9.57 | 6.24 | 6.81 | 3.18 | 1.90 | 1.85 | 3.78 | 6.13 | 2.72 | 2.04 | 1.94 | 3.51 |
| hsa-miR-6088 | 1.15E-04 | 7.25 | 2.65 | 2.84 | 5.34 | 5.71 | 4.97 | 5.76 | 5.77 | 5.58 | 6.21 | 3.01 | 4.13 | 1.81 | 1.87 | 1.79 | 2.01 | 2.26 | 1.89 | 1.75 | 1.81 | 2.14 |
| hsa-miR-6089 | 2.92E-03 | 3.50 | 8.35 | 7.45 | 9.46 | 9.94 | 10.65 | 10.75 | 10.59 | 10.35 | 11.57 | 8.36 | 10.52 | 7.66 | 7.87 | 7.52 | 8.13 | 8.20 | 7.31 | 7.43 | 7.31 | 7.60 |
| hsa-miR-6090 | 1.84E-05 | 22.72 | 7.69 | 8.12 | 13.07 | 10.63 | 9.88 | 9.85 | 10.57 | 10.96 | 10.98 | 7.63 | 7.58 | 4.03 | 6.08 | 3.85 | 7.20 | 7.22 | 4.49 | 5.78 | 3.45 | 7.02 |
| hsa-miR-6124 | 4.71E-05 | 3.13 | 3.08 | 5.15 | 4.26 | 3.82 | 3.09 | 3.96 | 4.17 | 3.85 | 4.67 | 2.82 | 3.27 | 2.55 | 2.00 | 2.16 | 2.25 | 1.96 | 2.69 | 2.24 | 2.01 | 2.37 |
| hsa-miR-6125 | 9.48E-03 | 2.61 | 7.46 | 6.04 | 6.09 | 6.75 | 7.28 | 8.77 | 8.76 | 7.07 | 8.75 | 7.50 | 7.66 | 5.63 | 6.44 | 4.12 | 7.48 | 7.37 | 3.93 | 4.80 | 4.50 | 6.17 |
| hsa-miR-630 | 9.55E-04 | 8.64 | 4.37 | 2.38 | 6.49 | 6.48 | 5.98 | 8.00 | 8.72 | 6.39 | 8.42 | 4.74 | 5.85 | 1.87 | 1.77 | 1.66 | 4.46 | 5.84 | 1.70 | 1.83 | 1.75 | 2.22 |
| hsa-miR-638 | 7.29E-03 | 3.28 | 6.90 | 3.21 | 5.27 | 6.42 | 6.66 | 7.77 | 7.95 | 5.72 | 8.39 | 7.09 | 7.14 | 3.92 | 3.84 | 1.94 | 6.85 | 6.56 | 2.13 | 2.09 | 2.05 | 3.78 |
| hsa-miR-6510-5p | 4.55E-04 | 3.25 | 4.73 | 6.97 | 6.13 | 5.37 | 5.72 | 5.18 | 5.06 | 6.03 | 6.03 | 6.47 | 6.54 | 3.87 | 2.61 | 2.19 | 4.21 | 4.57 | 3.15 | 1.95 | 1.88 | 3.19 |
| hsa-miR-6716-3p | 1.69E-03 | 0.38 | 5.71 | 6.22 | 2.13 | 4.27 | 5.44 | 3.80 | 3.96 | 4.57 | 3.03 | 5.30 | 5.45 | 6.59 | 7.12 | 5.81 | 6.42 | 5.80 | 5.97 | 6.28 | 6.52 | 5.99 |
| hsa-miR-6797-3p | 1.38E-03 | 0.45 | 4.74 | 4.66 | 3.05 | 3.30 | 4.61 | 3.39 | 3.07 | 3.99 | 2.89 | 5.95 | 4.28 | 5.67 | 5.16 | 5.83 | 5.91 | 5.91 | 5.55 | 5.09 | 5.16 | 5.36 |
| hsa-miR-6800-3p | 3.81E-05 | 0.35 | 4.05 | 3.19 | 2.50 | 2.28 | 2.82 | 2.53 | 2.31 | 2.63 | 2.21 | 4.44 | 3.76 | 5.15 | 3.99 | 4.93 | 4.82 | 4.82 | 5.15 | 3.86 | 4.69 | 4.46 |
| hsa-miR-6800-5p | 1.56E-03 | 3.39 | 7.19 | 5.49 | 6.35 | 6.71 | 7.26 | 8.26 | 8.57 | 7.05 | 8.63 | 7.09 | 7.35 | 4.13 | 5.35 | 4.01 | 6.82 | 6.76 | 4.62 | 3.84 | 3.28 | 5.58 |
| hsa-miR-6821-5p | 4.50E-06 | 11.17 | 5.26 | 5.52 | 8.61 | 6.44 | 6.51 | 6.65 | 7.94 | 6.88 | 8.48 | 6.26 | 6.11 | 2.06 | 2.04 | 1.97 | 4.31 | 4.40 | 1.97 | 1.76 | 1.87 | 2.49 |
| hsa-miR-6869-5p | 2.69E-04 | 5.73 | 6.74 | 4.50 | 7.71 | 7.07 | 7.04 | 7.82 | 8.36 | 6.89 | 8.54 | 6.92 | 7.01 | 2.36 | 2.03 | 1.82 | 6.53 | 5.79 | 1.88 | 1.71 | 1.73 | 3.10 |
| hsa-miR-6879-5p | 4.95E-04 | 0.34 | 5.68 | 6.74 | 3.62 | 4.76 | 3.86 | 4.63 | 4.34 | 3.87 | 4.02 | 2.44 | 5.71 | 6.93 | 6.74 | 6.68 | 5.74 | 4.87 | 6.46 | 6.88 | 6.62 | 6.49 |
| hsa-miR-6889-3p | 8.86E-07 | 0.39 | 3.44 | 2.51 | 2.19 | 2.41 | 2.73 | 2.27 | 2.30 | 2.40 | 2.16 | 3.06 | 3.44 | 3.99 | 3.54 | 4.14 | 3.76 | 3.52 | 4.05 | 4.68 | 4.41 | 3.55 |
| hsa-miR-7107-5p | 2.35E-05 | 4.16 | 5.68 | 6.83 | 7.38 | 6.01 | 7.04 | 5.98 | 6.71 | 6.43 | 7.85 | 6.58 | 6.28 | 4.73 | 4.76 | 3.45 | 4.62 | 5.44 | 3.64 | 3.28 | 3.27 | 4.60 |
| hsa-miR-7110-5p | 3.49E-03 | 2.51 | 5.41 | 7.09 | 6.44 | 5.70 | 6.33 | 5.85 | 5.38 | 6.29 | 6.44 | 6.82 | 6.92 | 5.21 | 3.87 | 2.11 | 4.98 | 5.75 | 4.37 | 2.13 | 2.32 | 3.99 |
| hsa-miR-7641 | 2.57E-04 | 58.34 | 3.89 | 7.16 | 10.69 | 8.55 | 6.31 | 8.55 | 10.12 | 8.23 | 8.94 | 2.10 | 3.43 | 1.79 | 1.72 | 1.70 | 3.48 | 3.48 | 1.81 | 3.20 | 2.26 | 4.13 |
| hsa-miR-7704 | 3.70E-03 | 2.15 | 4.08 | 2.67 | 4.07 | 4.28 | 3.84 | 4.87 | 4.53 | 4.21 | 4.77 | 4.29 | 4.54 | 2.00 | 1.83 | 1.71 | 4.34 | 4.28 | 1.76 | 1.69 | 1.74 | 2.39 |
| hsa-miR-7975 | 1.68E-04 | 5.32 | 6.80 | 8.53 | 9.45 | 9.18 | 6.84 | 5.70 | 8.55 | 7.37 | 7.85 | 6.75 | 5.64 | 5.20 | 5.04 | 6.64 | 4.45 | 4.39 | 4.69 | 4.54 | 7.60 | 4.50 |
| hsa-miR-7977 | 6.72E-06 | 19.31 | 6.16 | 9.11 | 9.79 | 10.22 | 6.83 | 6.36 | 9.16 | 8.55 | 9.05 | 6.96 | 5.72 | 4.19 | 4.21 | 4.97 | 4.85 | 3.92 | 4.11 | 3.81 | 4.70 | 3.42 |
| hsa-miR-8069 | 6.43E-05 | 10.03 | 6.91 | 6.59 | 10.06 | 8.88 | 7.17 | 9.09 | 9.51 | 7.83 | 9.60 | 6.84 | 7.00 | 5.38 | 3.67 | 2.81 | 6.37 | 6.01 | 2.31 | 5.08 | 2.62 | 5.54 |
| hsa-miR-940 | 3.59E-04 | 0.39 | 5.45 | 4.99 | 3.35 | 3.92 | 4.66 | 4.43 | 3.32 | 4.13 | 3.64 | 6.36 | 5.78 | 6.22 | 5.14 | 6.46 | 6.52 | 6.52 | 6.46 | 5.83 | 5.61 | 6.00 |

* Differential expression of miRNAs was analyzed by Student's t-test. A value of *P*< 0.05 (fold change ≥2 or ≤0.5) was regarded as statistically significant. LM, leptomeningeal metastasis; BM, brain metastasis; NC, non cancer.

**Supplemental Table 2 The mappable miRNAs differentially expressed in matched CSF samples from LM patients** at diagnosis and after initial therapy

| **Systematic**  **Name** | ***P* value*** | **Fold Change** | **After initial therapy** | | | | | | **At diagnosis** | | | | | |
| --- | --- | --- | --- | --- | --- | --- | --- | --- | --- | --- | --- | --- | --- | --- |
|  |  |  | **Post. 1** | **Post. 2** | **Post. 3** | **Post. 4** | **Post. 5** | **Post. 6** | **Pre. 1** | **Pre. 2** | **Pre. 3** | **Pre. 4** | **Pre. 5** | **Pre. 6** |
| hsa-miR-4800-5p | 7.81E-03 | 0.46 | 3.92 | 4.14 | 2.43 | 2.33 | 3.75 | 2.82 | 4.40 | 4.43 | 3.99 | 4.48 | 5.16 | 4.42 |
| hsa-miR-7975 | 2.60E-03 | 0.34 | 3.91 | 4.56 | 3.10 | 4.39 | 5.80 | 3.65 | 4.82 | 5.04 | 4.65 | 6.52 | 7.43 | 5.66 |
| hsa-miR-7977 | 2.50E-03 | 0.39 | 5.01 | 4.79 | 4.25 | 5.58 | 6.79 | 4.99 | 5.73 | 5.27 | 5.20 | 6.80 | 8.49 | 6.61 |

* Differential expression of miRNAs was analyzed by Paired t-test. A value of *P*<0.05 (fold change ≥2 or ≤0.5) was regarded as statistically significant. Pre, leptomeningeal metastases at diagnosis; Post, matched leptomeningeal metastases after initial therapy.
